# Computational GWAS Meta Meta Analysis Revealing Cross Talk Between Cannabis CNR1 and DRD2 Receptors Optimizing Long-Term Outcomes for Cannabis Use Disorder (CUD) By Enhancing Dopamine Homeostasis Promoting High-Quality Cannabis Medicinals

**DOI:** 10.21203/rs.3.rs-8140327/v1

**Published:** 2025-11-19

**Authors:** Kenneth Blum, Alireza Sharafshah, Jag Khalsa, Kai-Uwe Lewandrowski, Panayotis K Thanos, Marco Lindeau, Álvaro Dowling, Rafaela Dowling, Jao Paulo Bergamaschi, Albert Pinhasov, David Baron, Catherine A. Dennen, Joseph P Morgan, Igor Elman, Eliot L. Gardner, Mark S. Gold, Edward J. Modestino, Fuehrlein Brian, Paul R. Carney, Rene Cortese, Abadalla Bowirrat, Keerthy Sunder, Kavya Mohankumar, Foojan Zeine, Nicole Jafari, Milan T. Makale, Debasis Bagchi, Mauro Ceccanti, Rossano K A Fiorelli, Sérgio Luís Schimidt, Daniel Sipple, Alexander P. L. Lewandrowski, Gianni Matare, Shaurya Mahajan, Yatharth Mahajan, Chynna Fliegelman, Colin Hanna, Rajendra D. Badgaiyan

**Affiliations:** 1 Department of Molecular Biology, Adelson School of Medicine, Ariel University, Ariel, Israel; 2 Division of Addiction Research & Education, Center for Sports, Exercise, and Mental Health, Western University of Health Sciences, Pomona, CA., USA; 3 Division of Clinical Neurology, The Blum Institute of Neurogenetics & Behavior, Austin, TX., USA; 4 Cellular and Molecular Research Center, School of Medicine, Guilan University of Medical Sciences, Rasht, Iran.; 5 Department of Medicine, University of Maryland, School of Medicine, Baltimore, MD., USA.; 6 Division of Personalized Pain Therapy Research & Education, Center for Advanced Spine Care of Southern Arizona, Tucson, AZ., USA; 7 Department of Orthopaedics, Fundación Universitaria Sanitas Bogotá D.C. Colombia.; 8 Department of Orthopaedics, Universidade Federal do Estado do Rio de Janeiro, Brazil; 9 Department of Surgery, Arizona University School of Medicine, Tucson, AZ., USA; 10 Behavioral Neuropharmacology and Neuroimaging Laboratory on Addictions, Clinical Research Institute on Addictions, Department of Pharmacology and Toxicology, Jacobs School of Medicine and Biosciences, State University of New York at Buffalo, Buffalo, NY, USA.; 11 Department of Psychiatry, Stanford University School of Medicine, Palo Alto, CA., USA; 12 Department of Family Medicine, Jefferson Health Northeast, Philadelphia, PA, USA; 13 Substance Use Disorders Institute University of Sciences, Philadelphia, PA, USA; 14 Department of Psychiatry and Cambridge Health Alliance, Harvard Medical School, Cambridge, MA., USA; 15 Neuropsychopharmacology Section, Intramural Research Program, National Institute on Drug Abuse, National Institutes of Health, Baltimore, Md., USA; 16 Department of Psychiatry, Washington University School of Medicine, St. Louis, MO, USA; 17 Department of Psychology, Curry College, Milton, MA, USA; 18 Department of Psychiatry, Yale University, New Haven, CT, USA; 19 Division Pediatric Neurology, University of Missouri, School of Medicine, Columbia, MO, USA.; 20 Department of Child Health, Child Health Research Institute, School of Medicine, University of Missouri, Columbia, MO, USA.; 21 Department of Medicine, University of California, Riverside School of Medicine, Riverside, CA, USA.; 22 Karma Doctors & Karma TMS, and Suder Foundation, Palm Springs, CA, USA.; 23 Division of Research, Ocean State Research Institute, Providence, RI, USA; 24 Awareness Integration Institute, San Clemente, CA, USA; 25 Department of Applied Clinical Psychology, The Chicago School of Professional Psychology, CA, USA; 26 Division of Personalized Medicine, Global Growth Institute, Inc., San Clemente, CA, USA; 27 Division of Personalized Medicine, Cross-Cultural Research and Educatiaonal Institute, San Clemente, CA, USA; 28 Department of Radiation Oncology, University of California San Diego, La Jolla, CA, USA; 29 Department of Pharmaceutical Sciences, Texas Southern University College of Pharmacy, Houston, TX, USA; 30 Alcohol Addiction Program, Latium Region Referral Center, Sapienza University of Rome, Roma, Italy; 31 Department of General and Specialized Surgery, Gaffrée e Guinle Universitary Hospital (EBSERH), Federal University of the State of Rio de Janeiro (UNIRIO), Rio de Janeiro, RJ, Brazil; 32 Programa de Pós-Graduação em Neurologia, Universidade Federal do Estado do Rio de Janeiro, Rio de Janeiro, Brazil.; 33 M Health Fairview University of Minnesota Medical Center, Minneapolis, MN.,USA; 34 Dornsife College of Letters, Arts and Sciences, University of Southern California, Los Angeles, CA., USA; 35 Department of Psychology, University South Dekoda, Vermillion, SD., USA.; 36 Department of Psychiatry, Mt. Sinai University, School of Medicine, New York, NY., USA

**Keywords:** Cannabis, Cannabis User Disorder, Hyperdopaminergia, CNR1 receptor, GARS, Pro-dopamine regulation (KB220)

## Abstract

This paper presents a shared perspective from scientists and clinicians seeking to harness the therapeutic potential of cannabis while addressing Cannabis Use Disorder (CUD) through reproducible scientific findings. Acute cannabis use may produce temporary well-being, but chronic use can create a “pseudo-feeling” of well-being, leading to tolerance, discomfort, and adverse effects. Rather than blocking CNR1 receptors, which may induce hypodopaminergia, we propose a pro-dopaminergic strategy using a natural nutraceutical formulation designed to enhance dopamine release and upregulate D2 receptor mRNA, thereby increasing D2 receptor density.

Historically, low-potency cannabis (2–4% Δ9-THC) was not associated with major neuroanatomical, psychotic, or depressive outcomes. However, modern cannabis potency has risen dramatically, now exceeding 17% Δ9-THC on average, with concentrates reaching up to 97%, potentially increasing the risk of dopamine dysfunction and CUD. Computational analyses identified the drd2 gene as central to cannabis pharmacology. Meta and Meta-Meta analyses refined a Primary and Secondary Gene List, leading to 23 final genes, including DRD2, DRD1, BDNF, GNAT1, POU3F2, and SLC67A4. Significant miRNAs (hsa-miR-15a-5p, hsa-miR-16–5p) and transcription factors (SP1, REST, EGR1) were also revealed, highlighting dopaminergic pathway involvement. Additional systems biology results indicated heroin dependence as the highest-risk manifestation linked to these genes, and PGx analyses suggested DRD1, DRD2, BDNF, and OPRM1 as promising targets for future studies.

Given the failure of CNR1 antagonists such as Rimonabant, we argue for an opposite approach: restoring dopamine balance through CNR1 stimulation rather than inhibition.

## Graphical Abstract

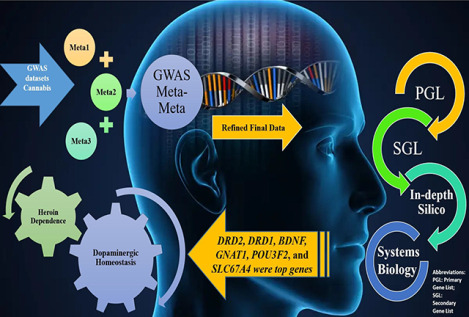

An overview on the whole process of study design from dataset preparation to candidate top-ranked genes and highly involved pathways in Cannabis.

## 1.0 Background and hypothesis

This paper represents the views of several scientists and clinicians who are focused on harnessing the therapeutic potential of cannabis while also addressing Cannabis Use Disorder (CUD). Based on neurochemical and genetic evidence, we propose a biphasic approach to addiction prevention and treatment, which can be applied to various dependencies, including cannabis, alcohol, nicotine, and glucose. (1) In the short term, the treatment should involve selective blocking of postsynaptic dopamine receptors (D1–D5) in the Nucleus Accumbens (NAc). Over the long term, however, sustained activation and release of dopamine (DA) at the NAc should be the goal, as failure to achieve this can lead to abnormal mood, behavioral disturbances, and even suicidal ideation (2). Individuals with genetic predispositions, such as low serotonergic or dopaminergic receptor availability and high synaptic DA catabolism, are more susceptible to self-medicating behaviors. This can include the misuse of substances such as cannabis, alcohol, opiates, psychostimulants, nicotine and compulsive behaviors like gambling or excessive internet use (2). While acute use of these substances may induce a temporary sense of well-being, prolonged abuse results in tolerance and subsequent negative effects, including disease and discomfort (3).

Individuals carrying the DRD2 A1 allele, which is associated with a lower number of DA receptors, are particularly prone to cannabis craving. Conversely, those with sufficient DA receptor levels exhibit lower craving behaviors (4). To mitigate substance abuse, one strategy could involve promoting DA D2 receptor proliferation in genetically susceptible individuals (5). Although in vivo experiments with typical D2 receptor agonists have shown downregulation, in vitro studies suggest that gentle, consistent stimulation of the DA receptor system through a D2 agonist can lead to significant receptor proliferation despite genetic predispositions.(6)

Cannabis use may affect the balance of dopamine signaling in the mesolimbic system, possibly leading to compensatory changes over time, such as D2 receptors mRNA expression modulation.(7) Instead of blocking CNR1 receptors, which can induce hypodopaminergia(8,9), we propose using a natural, pro-dopaminergic nutraceutical formulation to stimulate DA release. This approach may induce D2 receptor mRNA expression and promote receptor proliferation in humans.(10)

Clinical trials, including double- and triple-blind studies, have demonstrated that increasing D2 receptor density can significantly reduce craving behaviors.(11) This concept has been further validated through research showing compensatory overexpression of DRD2 receptors, which resulted in reduced alcohol craving in alcohol-preferring rodents.(12–14) Using natural dopaminergic therapies to promote long-term DA activation provides a promising, safe, and effective treatment strategy for Reward Deficiency Syndrome (RDS), which encompasses various disorders, including Substance Use Disorders (SUD), Attention Deficit Hyperactivity Disorder (ADHD), and obesity.(15)

This approach aligns with the understanding that dopamine in the NAc functions as a “wanting” signal within the mesolimbic DA system, playing a critical role in driving addictive and reward-seeking behaviors.(16–18) One important article related to benefits and risks of Cannabis and Cannabinoids in psychiatry recommended that there is a need for evidence-based, safe, and non-addictive CBD medications, and currently the evidence is insufficient to support the prescription of cannabinoids for the treatment of psychiatric disorders (19).

## 2.0 Brain Reward Circuitry is Regulated by Complex Interactions of Multiple Neurotransmitters

It is well established that brain reward circuitry is modulated by the interactions of various neurotransmitters, with dopamine (DA) release in the Nucleus Accumbens (NAc) playing a central role in this process. Dopamine is essential for regulating natural reward mechanisms, but the release of DA into NAc synapses involves a complex series of neural events. In 1989, our laboratory introduced the concept of an interconnected series of events within the mesolimbic system that result in the net release of DA, a process we termed the “brain reward cascade”(3). This model has since been updated and refined in 2024 (see Figure 1).

The mesolimbic system plays a central role in mediating feelings of reward and well-being (20). The “brain reward cascade” describes the sequence of neurochemical events that lead to this sense of reward, involving key pathways such as serotonergic, enkephalinergic, GABAergic, and dopaminergic. The synthesis, storage, release, and function of these neurotransmitters are governed by genetic factors, including mRNA-directed protein expression (21). It has been proposed that genetic research may enable personalized psychiatric treatments by identifying individuals’ specific neurochemical profiles (22).

### 2.1. Cannabis, Dopamine Dysregulation, and Reward Deficiency Behaviors

Dopamine (DA) plays a crucial role in various neurochemical processes and is associated with behaviors such as pleasure, stress reduction, and motivation. A deficiency in DA function, particularly within the mesolimbic system, can lead to an inability to experience pleasure (anhedonia) and difficulty managing stress, both of which are associated with addiction-related behaviors (23). Genetic predispositions that result in lower DA activity can make individuals more likely to engage in behaviors or substances, such as cannabis, that stimulate DA release in the mesolimbic system (24).

Chronic cannabis use, particularly during adolescence, has been linked to decreased DA release in the striatum, resulting in hypodopaminergia, poor memory, attention deficits, and impaired learning.(25) Studies using [18F]-DOPA PET imaging have demonstrated that long-term cannabis use is associated with reduced DA synthesis, diminished reward sensitivity, lower motivation, and apathy. Genetic factors, such as the presence of the 9/9 allele polymorphism, result in higher D2/D3 receptor availability compared to the 10/10 alleles, particularly in heavy, early-onset cannabis users.(26) Additionally, carriers of the DRD4 7R polymorphism are more likely to experiment with cannabis than non-carriers. Research indicates that chronic cannabis use correlates with reduced dopamine reactivity, particularly among users with higher negative emotionality scores.(27,28)

Evidence also suggests that high doses of Δ9-THC may increase DA release by inhibiting GABAergic activity in the Ventral Tegmental Area (VTA)(29), potentially heightening fear responses in cannabis users. Animal studies show that repeated administration of Δ9-THC induces depressive-like behaviors, prolonged anhedonia, and altered dopaminergic activity in the mesolimbic system due to CNR1 receptor impairment.(30) Moreover, individuals diagnosed with CUD are four times more likely to develop depressive symptoms, even if they have no initial history of depression.(31)

Historically, low-potency cannabis (2–4% Δ9-THC) was not linked to significant neuroanatomical changes, psychosis, or depression. However, the average Δ9-THC concentration in cannabis has risen dramatically, from 8.9% to 17.1% between 2008 and 2017.(32) Modern cannabis products, such as pastes, gummies, and e-vaping devices, can contain very high levels of Δ9-THC, increasing the risk of hypodopaminergia, memory deficits, attention problems, and learning impairments, particularly in chronic users and adolescents with CUD. The severity of brain changes and related symptoms appears to be closely linked to the potency, frequency, and duration of cannabis use.

A novel model (Figure 2) proposes a biphasic approach to addressing these issues: an initial short-term blockade, followed by long-term upregulation of dopamine activity using a pro-dopamine regulator, primarily targeting behaviors associated with Reward Deficiency Syndrome (RDS).(3,33,34)

Recent research by Thanos’ group has explored the relationship between the endocannabinoid system and dopamine-related reward deficiency behaviors, particularly in the context of cognitive impairment.(35) Fatty acid-binding protein 5 (FABP5) plays a critical role in the brain’s endocannabinoid system by facilitating the intracellular transport of anandamide (AEA) and Δ9-tetrahydrocannabinol (THC), the psychoactive component of cannabis. Their studies demonstrated that deletion of FABP5 resulted in cognitive deficits, which were reversed by THC inhalation. Additionally, FABP5-deficient mice showed altered pharmacokinetics of THC and elevated AEA levels following THC administration. This suggests that FABP5 is a key regulator of THC’s effects. Chronic administration of THC resulted in dose-dependent alterations in CNR1 receptor levels throughout the brain and influenced locomotor activity in open-field tests. Prenatal exposure to vaporized THC was associated with attention deficits and memory impairments, with evidence suggesting that prenatal THC exposure might lead to developmental issues such as low birth weight and subsequent obesity.(35–42)

Moreover, studies have identified FABP5 as a key factor in modulating nicotine place preference and its role in ethanol consumption, which appears to be regulated differently between males and females, potentially mediated by the stress response system. The interaction of FABP5 with substances like cannabinoids and behaviors such as gambling, sex, and excessive pleasure-seeking activities (e.g., video gaming) highlights its broad role in dopamine-mediated reward pathways.(40,43)

These substances and behaviors, particularly cannabis use, stimulate the release of dopamine into the synapses of the NAc, even during prenatal exposure.(44–49) Acute engagement in these behaviors may serve as a form of self-medication for individuals experiencing dopamine deficiency, temporarily alleviating their hypodopaminergic state through enhanced dopamine release.

### 2.2 “Pseudo Feeling“ of Well-Being

Self-medication through psychoactive substances and maladaptive behaviors often provides temporary relief from discomfort and a false sense of well-being.(50,51) However, chronic misuse of these substances and engagement in compulsive behaviors can disrupt the brain’s reward cascade. This disruption involves the inhibition of neurotransmitter synthesis, depletion of neurotransmitter storage, formation of toxic pseudo-neurotransmitters, and dysfunction of neurotransmitter receptors, both structurally and in terms of receptor density. Continued abuse results in further neurotransmitter dysregulation, leading to escalating, uncontrollable cravings as individuals seek to restore a “feel-good” response (FGR).

Research has shown that individuals with certain genetic polymorphisms are particularly vulnerable to the adverse effects of environmental and lifestyle stressors. These genetic variations heighten the risk of impulsive, compulsive, and addictive behaviors, including cocaine abuse.(52,53) The shared genetic predispositions that influence the brain’s natural reward pathways offer a framework for understanding the interconnectedness of these behaviors. This supports the concept of Reward Deficiency Syndrome (RDS), an umbrella term used to describe a spectrum of genetically influenced behaviors related to addiction, impulsivity, and compulsiveness.(54)

In this context, psychoactive substances and maladaptive behaviors are seen as potential candidates for addiction, driven by both genetic factors and environmental influences, such as availability and social pressures.(55) This model highlights how addiction develops as a result of the interaction between inherited vulnerabilities and external factors.

### 2.3 Cannabis-Induced Hypodopaminergic Anhedonia and Cognitive Decline in Humans

In recent years, cannabis use among young adults has significantly increased, leading to a rising prevalence of Cannabis Use Disorder (CUD), with an estimated rate of 8.3% in the United States. Studies indicate that cannabis exposure is linked to hypodopaminergic anhedonia (depression), cognitive decline, memory deficits, inattention, impaired learning, reduced dopamine response, heightened emotional dysregulation, and increased addiction severity among young adults.(56,57) The addiction medicine community is particularly concerned about the high levels of Δ9-THC present in modern cannabis products, such as edibles and vaping devices. These high-THC products may exacerbate cognitive impairments and mental health issues in young adult users.(38,58)

Emerging research suggests that restoring dopamine homeostasis through upregulation therapies may help normalize behavior in chronic cannabis users suffering from cannabis-induced hypodopaminergic anhedonia and cognitive dysfunction.(11) Such research in the psychological, neurobiological, genetic, and epigenetic domains could inform policies on the decriminalization of recreational cannabis use.

The concern about the high THC content in cannabis products (up to 90% THC in some edibles and vaping products) is growing within the addiction medicine field. This high potency may worsen symptoms of hypodopaminergic anhedonia and cognitive decline in long-term users.(59) Additionally, serious respiratory and pulmonary issues, including chronic obstructive pulmonary disease (COPD), have been reported among users of e-vaping devices.(58,60)

### 2.4 Cannabis and Neuroanatomic Alterations and Cognition

Cannabidiol (CBD) has been shown to mitigate the harmful effects of THC and may protect the brain from damage, potentially through CNR1 receptor antagonism.(61) The adverse effects of THC include dose-dependent psychotic, cognitive, and behavioral symptoms.(60) Several structural neuroimaging studies have documented that chronic cannabis use is associated with reduced gray matter volumes, particularly in areas such as the medial temporal cortex, orbitofrontal cortex, temporal poles, parahippocampal gyrus, and insula. Additionally, neuroanatomical alterations have been observed in the medial temporal and frontal cortex, cerebellum(62), fusiform gyrus, temporal pole, superior temporal gyrus, and occipital cortex.(63)

One of the primary concerns, especially in young adults with developing brains, is the detrimental impact of high doses of Δ9-THC on cognitive functions. Neuroanatomical changes in the prefrontal-hippocampal regions and the subsequent downregulation of CNR1 receptors have been linked to cognitive impairments, including deficits in working memory, decision-making, and inhibitory control in chronic cannabis users.(63–65) The CNR1 receptors, critical to processes involving motivation, emotion, and affect regulation, are highly concentrated in these brain areas. Upregulation of CNR1 receptors may help counteract THC-induced brain damage. Fortunately, cognitive function may recover after 4–6 weeks of cannabis abstinence.(66,67)

In adult cannabis users, brain activation patterns show a decrease in areas such as the middle temporal gyrus, insula, and striate cortex and an increase in regions like the superior and posterior transverse temporal gyri, inferior frontal gyrus, and middle temporal gyrus. In contrast, adolescent cannabis users display increased activation in the inferior parietal gyrus and putamen compared to healthy controls.(68) These functional changes suggest neuroadaptive processes in cannabis users that may serve as compensatory mechanisms.(68)

### 2.5 Epigenetics of Cannabis

The first evidence of epigenetic effects from prenatal THC exposure was reported by Blum’s group in 1980(69), demonstrating that perinatal exposure to delta-9-THC in mice altered enkephalin and norepinephrine sensitivity in the vas deferens of the F1 generation. This finding was later confirmed by Spano and Hurd’s team(70), who observed that THC-exposed rats displayed increased heroin-seeking behavior, shorter latency to initiate actions, and heightened heroin response under stress. These effects were associated with reduced preproenkephalin (PENK) mRNA expression in the nucleus accumbens during early development, followed by increased expression in adulthood, particularly in the amygdala.(71)

Furthermore, research on social isolation in rats revealed that five weeks of isolation led to a selective reduction in mRNA levels for fatty acid amide hydrolase (FAAH) and cannabinoid receptor type 1 (Cnr1) genes in the amygdala and prefrontal cortex, driven by epigenetic modifications such as histone acetylation and methylation.(72) In another study, chronic cocaine administration increased Cnr1 expression in several brain regions, including the prefrontal cortex, nucleus accumbens, hippocampus, and amygdala. Alterations in endocannabinoid levels were also observed, with chromatin immunoprecipitation revealing enrichment of activating histone markers at specific endocannabinoid genes in the hippocampus following cocaine intake.(73)

Additionally, in a study on activity-based anorexia (ABA), rats displayed downregulation of the Cnr1 gene in the hypothalamus and nucleus accumbens (NAc), accompanied by increased DNA methylation at the gene promoter in the NA.(74) The endocannabinoid system (ECS) is crucial for regulating stress responses, with 2-arachidonoylglycerol (2-AG) limiting glutamate release via CNR1 receptor activation. However, chronic stress can desensitize CNR1 receptors due to 2-AG overstimulation. The protective role of 2-AG in stress resilience has been highlighted, with evidence suggesting that epigenetic regulation, specifically through Lysine Specific Demethylase 1, plays a key role in ECS function.(75)

Some animal studies suggest that CNR1 antagonists could be a therapeutic target for various reward-related behaviors, including cannabis use disorder, nicotine dependence, binge alcohol consumption, cognitive impairment, obesity, and substance use disorders, among others.(8,76–89)

While animal models have contributed significantly to understanding the pharmacological and molecular mechanisms of disease, the translation to human therapeutic interventions requires caution. We propose that blocking CNR1 receptors may offer short-term benefits but advocate for long-term CNR1 receptor activation to align with natural physiological processes. With no FDA-approved cannabis treatment and increasing cannabis use in the U.S., which has the highest prevalence globally (17.9% in individuals aged 12 and older), the potential for increased cannabis abuse is substantial. In 2020, 5.1% of Americans were estimated to have Cannabis Use Disorder (CUD).(5)

Genetic and epigenetic factors play a significant role in both cannabis use and the development of CUD. Research suggests that 50–70% of CUD liability and 40–48% of cannabis use initiation are genetically influenced. Additionally, genetic liability for CUD has a strong correlation with schizophrenia, beyond the influence of tobacco or cannabis use alone. High-THC cannabis products (up to 90% THC in waxes) have been associated with increased risk of psychosis in individuals with CUD.(5,90)

### 2.6 Blocking CNR1 Receptors with Pregnenolone : A Cautionary Note

The legalization of recreational cannabis in the United States, much like the earlier legalization of alcohol, has led to a rise in Cannabis Use Disorder (CUD). Currently, there is no FDA-approved treatment for the abuse and addiction associated with high-potency THC. This has placed significant pressure on the National Institute on Drug Abuse (NIDA) and its director, Nora Volkow, to find an effective solution.(91) The pharmaceutical industry, despite its interest in addressing the issue, remains uncertain about the best approach to develop a successful treatment for CUD.

There are two main competing perspectives on how to approach CUD treatment. The first advocates for blocking CNR1 receptor activity to reduce dopamine release in the nucleus accumbens, aiming to induce extinction of drug-seeking behavior. The second approach proposes gently activating CNR1 receptors to promote dopamine homeostasis. While the first method may offer some short-term benefits, it risks long-term adverse effects, such as depression and suicidal ideation (SI).(92)

In adolescents, there is evidence of hyperdopaminergia in the developing brain, which may intensify their drive to experience heightened euphoria from cannabis use.(93) However, our laboratory has shown that adolescents with RDS, particularly those from mixed-gender cohorts with RDS-affected parents, are at an increased risk for addictive behaviors. Among these individuals, 95% demonstrated a predisposition toward drug-seeking, and 64% toward alcohol-seeking behaviors, indicative of a hypodopaminergic state.(94)

### 2.7 Can We Find a Solution to Treat CUD?

In a search for potential treatments for cannabis abuse and intoxication, Vallée et al. (2014) proposed that pregnenolone, a precursor to all steroid hormones, may be useful as an anti-cannabis agent. Δ9-Tetrahydrocannabinol (THC), the primary psychoactive component of cannabis, increases pregnenolone synthesis in the brain by stimulating the CNR1 receptor. Pregnenolone, in turn, acts as a specific inhibitor of CNR1 receptor signaling, reducing the effects of THC. While this mechanism provides a plausible basis for using pregnenolone to counteract cannabis intoxication, we offer an alternative view.

Pregnenolone has been shown to inhibit CNR1 receptor activity, thereby diminishing the effects of THC. Although this may appear promising, we argue that Vallée et al. (2014) may have overestimated the potential of CNR1 receptor blockade as a long-term solution to cannabis intoxication and addiction. Notably, other CNR1 receptor antagonists, such as Rimonabant, were withdrawn from the world-market and rejected by the FDA due to significant mood disturbances, including suicidal ideation (SI). Blocking CNR1 receptors can also reduce dopamine release by disinhibiting GABAergic signaling, which could lead to a hypodopaminergic state and heighten the risk of developing substance and behavioral addictions over time.

While pregnenolone is primarily considered an inactive steroid precursor, recent studies suggest it may protect the brain from cannabis intoxication. Neurosteroids, including pregnenolone, are synthesized in the brain and play a role in modulating the endocannabinoid system. THC has been shown to enhance pregnenolone synthesis by stimulating CNR1 receptors, with pregnenolone then acting as an allosteric modulator that inhibits CNR1 receptor activity without affecting THC’s affinity for the receptor.(95,96) This inhibition reduces THC-induced dopamine release in the nucleus accumbens (NAc) and attenuates THC-induced food intake, as demonstrated in studies involving CNR1 agonist WIN55,212–2.(97)

Although some researchers have argued for CNR1 receptor blockade as a treatment strategy for Cannabis Use Disorder (CUD) or THC toxicity(9,98–104), we contend that this approach should only be considered as a short-term intervention, especially in cases of THC-induced psychosis. Long-term CNR1 blockade, however, may have deleterious effects, including mood disturbances and the risk of SI.

Interestingly, the sulfated form of pregnenolone (pregnenolone sulfate) has been shown to enhance dopamine release, particularly in response to morphine in the rat NAc.(105) Pregnenolone sulfate significantly increases dopamine release at picomolar concentrations through an NMDA receptor-dependent mechanism, suggesting that its anti-craving effects may not be linked to CNR1 receptor blockade but rather to dopamine modulation. This raises questions about the true mechanism behind pregnenolone’s effects, which remain unresolved and require further investigation.(106)

Given the uncertainties surrounding pregnenolone’s role and the risks associated with anti-reward interventions like CNR1 blockade, more research is needed. The growing threat of cannabis intoxication in toddlers and children, along with concerns about cannabis use during pregnancy, nursing, and adolescence, underscores the urgency for the medical and scientific community to develop effective treatment strategies. Rather than relying on CNR1 antagonism, it may be more prudent to explore dopamine agonists for long-term treatment, such as the pro-dopamine regulator KB220, which has shown promise in balancing brain dopamine in clinical trials.(10,11,107,108)

## 3.0 Future Perspectives

Currently, with the increasing global prevalence of Cannabis Use Disorder (CUD), particularly in the United States, two key issues remain unresolved: the therapeutic potential of the cannabis plant and its numerous cannabinoids, and how to effectively address the growing CUD crisis. While this crisis does not carry the same immediate lethality as the opioid epidemic or the global problems of psychostimulant and alcohol abuse, it is nonetheless a significant public health concern.

The absence of FDA-approved treatments for CUD underscores the complexity of this issue. Despite ongoing research, a definitive solution remains elusive. At present, we do not claim to have answers to this multifaceted problem but aim instead to stimulate interest and exploration among leading scientific minds in both basic and clinical research. To achieve meaningful progress in addressing CUD, there is a need for open-mindedness and a commitment to rigorous, innovative research. This will be essential not only for developing safe and effective medicinal uses of cannabis but also for devising strategies to mitigate cannabis-seeking behaviors.(109–111)

### 3.1 Cannabis As Medicinals

The therapeutic value of the cannabis plant and its numerous cannabinoids has been a topic of considerable debate, with many states and countries either passing, proposing, or considering legislation to allow the use of cannabinoids—particularly cannabidiol (CBD)—as treatments for various clinical conditions, often without regulatory approval. To address this issue, we conducted a review of the published literature from the past 30-plus years using PubMed and Google Scholar databases, focusing on the use of cannabinoids as medical treatments. Here, we assess whether sufficient clinical evidence exists from well-designed studies and trials to justify the use of CBD or other cannabinoids as medicines.(38,39,112)

### 3.2. Recent Findings

Recent research indicates that while CBD and other cannabinoids hold promise, there is insufficient clinical evidence to formally recommend them as treatments for a broad range of conditions, despite widespread claims to the contrary. The few exceptions include the approved use of CBD for two rare forms of childhood epilepsy and the combination of CBD and THC for treating spasticity related to multiple sclerosis. Basic science suggests that CBD and other cannabinoids have the potential to treat multiple clinical conditions, but more rigorous preclinical and clinical studies, following regulatory guidelines, are necessary before broader medical applications can be endorsed. This will also require consistent breeding practices to ensure standardized THC content.

In most medicinal plants, a single primary pharmacologically active compound is responsible for the therapeutic effects—such as nicotine in the tobacco plant, cocaine in *Erythroxylon coca*, or morphine in ‘*Papaver somniferum’*. However, *Cannabis sativa* is unique in that it contains multiple pharmacologically active constituents with potential therapeutic benefits.(113) Among the 125 identified cannabinoids, only two—THC and CBD—have been extensively studied for their pharmacological and therapeutic effects. Nonetheless, other cannabinoids also demonstrate pharmacological activity and may hold therapeutic potential.

Several cannabis-related or cannabis-like products exhibit activity via CNR1 and/or CB2 receptors, but only four products have been approved for therapeutic use. Some other products have been withdrawn due to lack of efficacy or significant adverse reactions (Table 1). Further research and clinical trials are essential to better understand the safety and efficacy of these compounds.

Synthetic THC formulations, such as Marinol and Nabilone, have been approved for medical use in treating chemotherapy-induced nausea and vomiting and as appetite stimulants for patients with AIDS. Additionally, THC combined with CBD, marketed as Sativex, is approved for treating multiple sclerosis (MS)-associated spasticity in several countries, though not in the United States. Despite these approvals, THC’s addictive potential and adverse side effects make it less desirable for further pharmaceutical development.

Among cannabinoids, cannabidiol (CBD) has garnered significant attention and is one of the most extensively studied. Its approval for treating two rare forms of epilepsy—Lennox-Gastaut and Dravet syndromes in children—highlights its clinical potential. However, aside from these epilepsy indications and despite being promoted as a remedy for numerous other conditions in an industry valued at $20 billion annually, CBD has not been approved for treating other clinical conditions. Research does suggest that CBD holds promise for further therapeutic development, but well-structured clinical trials are needed for each specific condition.(112) Ongoing trials are addressing these gaps (https://clinicaltrials.gov).

Similarly, other cannabinoids, as reviewed by Khalsa et al.(112), also show potential but require further investigation. Cannabichromene (CBC), for example, could be studied for seizure treatment due to its neuroinflammatory properties and for inflammatory skin conditions like allergic dermatitis due to its effects on cytokines. Cannabidivarin (CBDV), with its neuroinflammatory and anti-inflammatory properties, could also be evaluated for seizure treatment and for acne management by targeting sebocytes. Tetrahydrocannabivarin (THCV), like CBD, shows promise for addressing a broad spectrum of conditions, including schizophrenia, epilepsy, obesity, neuropathy, retinopathy, nausea, pain, and skin conditions like dermatitis and acne, thanks to its antipsychotic, anti-inflammatory, and immunomodulatory effects.(114)

Cannabigerol (CBG), known for its antioxidant, anti-inflammatory, and gene-modulating effects, may be useful in treating dermatitis, acne, colorectal cancer, and colitis. These cannabinoids hold therapeutic potential for a wide array of conditions, and several clinical trials investigating them are currently in progress, as outlined in Table 2 (https://clinicaltrials.gov).

### 3.3. GWAS Meta-Meta analysis and new findings

#### Material and Methods

##### Extraction and Preparation of raw Data

GWAS catalog (https://www.ebi.ac.uk/gwas/home) was applied for mining raw data and we searched for Cannabis as a keyword and found three related Catalog IDs (CIDs). The initial datasets were EFO_0007585 for Cannabis Use, EFO_0007191 for Cannabis Dependence, and EFO_0008457 for Cannabis Dependence Measurement. GWAS Meta-analyses and GWAS Meta-Meta analysis were successfully performed on different Cannabis GWAS datasets via the Comprehensive Meta-Analysis version 3 (CMA3) tool.

Specifically, computational analyses involving the mapped genes found by various GWAS in each dataset were primarily addressed by a separate GWAS Meta-Analysis. In fact, these mapped genes along with a list of mapped genes from all datasets were then validated by a GWAS Meta-Meta Analysis. In summary, we choose 3 datasets through various Catalog IDs (CIDs). Each CID included both single and multiple GWAS studies resulting in a more final dataset. Meta-Meta analyses related to both *Cannabis Use disorder and Cannabis Dependence* were also employed to identify the significance and cumulative effect size (ES) based on the Partial Coefficient Correlation (PCC) using Fisher’s z transformation [115]

In essence, using inclusion/exclusion criteria after performing separate GWAS Meta analyses for each GWAS dataset in a CMA via CMA3 tool, we precisely obtained 3 separate meta-data, Meta1, Meta2, and Meta3. Specifically, the various types of inclusion criteria were as follows: unique GWAS dataset, published articles, GWA studies with a specific number of subjects, p-value < 5E-08 in accordance with GWAS consensus threshold, having OR/Beta (CI95%) scores, articles which reported mapped genes and their related SNPs. It is to be noted and elucidated herein that the selection of p-values utilized in our analysis required a two-phase procedure. First, all of the data in the GWAS datasets with p-values lower than 5E-08 remained and the rest of data with p>5E08 were discarded; in the next phase, among the SNPs of the remaining studies, the weakest p-values (which should be less than 5E-08) were selected to be included as a candidate for the meta-analysis. Importantly and for clarity, it is to be noted that in studies having multiple SNPs in the dataset, the included OR value in CMA3 from a respective study was considered based on the weakest (the most cautious) p-value of that SNP. Conversely, the exclusion criteria were as follows: duplicated studies, unpublished articles (Pre-Print or under review formats), p-value> 5E-08, missing OR/Beta and CI95%, articles without specific mapped genes and unspecified SNPs. Moreover, in terms of both our Meta-analysis and Meta-Meta Analysis of relevant GWAS data utilizing the CMA3 tool, which was based on an adjusted effect size data, we engaged in the following directive path : two groups or correlation >Dichotomous > unmatched groups > p-value and sample size for overall correlative outcome data. Finally, the entry data were as follows: total sample size, best reported p-values, 2-tailed, and positive effect direction (due to the nature of GWAS associations).

Following performing the GWAS Meta and GWAS Meta-Meta Analyses, all significant mapped genes were extracted from all datasets and incorporated into a single list. This Pre-Primary Gene List (PrePGL) consisted of a large file (raw data) including duplications, pseudogenes, RNA genes, and protein-coding genes. As such in a final attempt for refining the resultant meaningful gene list, we then deleted duplications, RNA genes and pseudogenes. Subsequent to this arduous ending step we obtained a refined list of protein-coding genes. Other analyses were then performed on this final refined file (PGL). All details and references are listed in Table 1.

#### GWAS Meta, GWAS Meta-Meta, in-depth silico, and Systems Biology Analyses

Notably, this novel investigative methodological strategy has been recently replicated and published in a number of prestigious journals on our prior published papers [121–124]. To summarize, by initially (first-step) utilizing GWAS Meta and GWAS Meta-Meta Analyses, that provided the requires statistical support to engage in the next few steps induced confidence to proceed. It is noteworthy to point out, to our current knowledge, the GWAS Meta-Meta analyses procedure, which appears not to be found in the literature (PubMed + Google scholar), suggesting a novel strategy in the computational AI neuroscience genomic field.

To reiterate for clarity, our strategy contains supplementary in-depth silico investigations, systems biology analyses, and Pharmacogenomics (PGx) approaches. We performed Protein-Protein Interactions (PPIs) utilizing a STRING-MODEL to help identify the interconnected members (Proteogenomics). Additionally, we employed an Enrichment Analysis to check the precision of the refined comprised genes in PGL. This strategy helped us find the most interconnected genes as potential candidates linked to cannabis us or misuse. Subsequently, following this lime of investigation we developed what we term a new *“Secondary Gene List “*(SGL).

Therefore, the *“Secondary Gene List “*(SGL), further defined a more in-depth silico analyses, and obviated another PPIs by STRING-MODEL, GMIs and TF-miRNA co-regulatory interactions (TF-miR CoRegIs) vilifying a novel NetworkAnalyst [using R package]. This further resulted in the adoption of an Enrichment Analyses through Enrichr. Systems Biology that was achieved via subsequent pathway (s), ontology, and Disease-Drug Analysis (See details in Table 1).

All relevant details and the strategy of analysis are summarized in Table 1. To enhance comprehension of this complex methodology, we provide herein, Figure 1 as an illustrated flowchart utilized in this investigation.

#### Results

##### GWAS Meta-Analysis and Meta-Meta Analysis Results

A total of 258 associations, 13 studies from various ethnicities were extracted from the 3 GWAS traits (Cannabis Use, Cannabis Dependence, and Cannabis Dependence Measurement) as the raw data. After preparation of data for GWAS Meta analyses, the results indicated that there were 7 unique studies and 1,190,454 subjects from Meta1, 5 unique studies and 568,944 subjects from Meta2, and finally, 1 study and 14,754 subjects from Meta3 (Table 2).

Our Meta-analysis results for each Meta group were as follows: Meta1 showed a significant association in a random model with a p-value lower than 6.5E-08, Z-value of 5.41, Fisher’s z transformed of 0.028 [0.018–0.039] (Supplementary Figure 1); Meta2 designated a significant association in a random model of effect size (p-value= 1.48E-5; Z-value= 4.33; Fisher’s z transformed = 0.023 [0.013–0.034]) (Supplementary Figure 2); and Meta3 indicated a significant random model of effect size (p-value=2E-08; Z-value= 5.613; Fisher’s z transformed = 0.046 [0.030–0.062]) (Supplementary Figure 3). Forest plots of all Meta1-3 analyses can be found as the supplementary Figures 1–3.

Following this calculation and subsequent results, we further performed a Meta-Meta analysis. This was accomplished by combining all three Meta groups (Meta1-Meta3). Interestingly, we discovered a significant association among these Meta groups (p-value= 1.69E-03; Z-value= 3.13; Fisher’s z transformed = 0.012 [0.004–0.020]. Figure 2 designates the Meta-Meta Analysis results in a Forest Plot with Favored regions (Fisher’s z transformed with 95% CI) and relative weights.

To check the publication bias, a Funnel Plot of the final Meta-Meta Analyses were generated (Figure 3) and extra details about the statistics of the performed Meta-Meta Analyses is summarized in the Table 3.

##### Protein-Protein Interactions (PPIs)

As mentioned in the method section, intergenic, non-coding transcript, and synonymous variants were ruled out, along with pseudogenes and RNA-coding genes. According to an acceptable threshold in GWAS accord (p-value<5E-08), 56 genes were included for the further steps; of note, 10 genes of the GARS SNPS were added to the refined list for checking the “dopamine homeostasis” impact involving reward deficiency known behaviors (e.g. RDS)RDS) which resulted in a total of 66 genes that were considered in the PGL. STRING-MODEL of PPIs showed that 43 of these 66 genes were unconnected and 23 genes remained in the primary PPI step. Specifically, STRING-MODEL scores were as follows: PPI enrichment p-value< 1.0e-16; average node degree= 1.94; average local clustering coefficient= 0.351] (Figure 4).

#### Secondary Protein-Protein Interactions (PPIs)

The secondary gene list (SGL) consisted of 23 combined genes; interestingly, *DRD2* was present in both Cannabis and GARS groups. Lastly, following two-step PPIs by STRING-MODEL, we found 23 completely connected genes resulting from the PGL (66 genes). Furthermore, STRING-MODEL scores were as follows: PPI enrichment p-value= 1E-16; average node degree=4.96; average local clustering coefficient= 0.644. To be clear, the remaining analyses described herein will be based on the SGL (23 genes). Employment of SGL enabled us to garnish a deeper view of plausible associations between dopamine homeostasis [or GARS impact] and Cannabis Dependence.

#### Gene-miRNA Interactions

**Figure 5.**
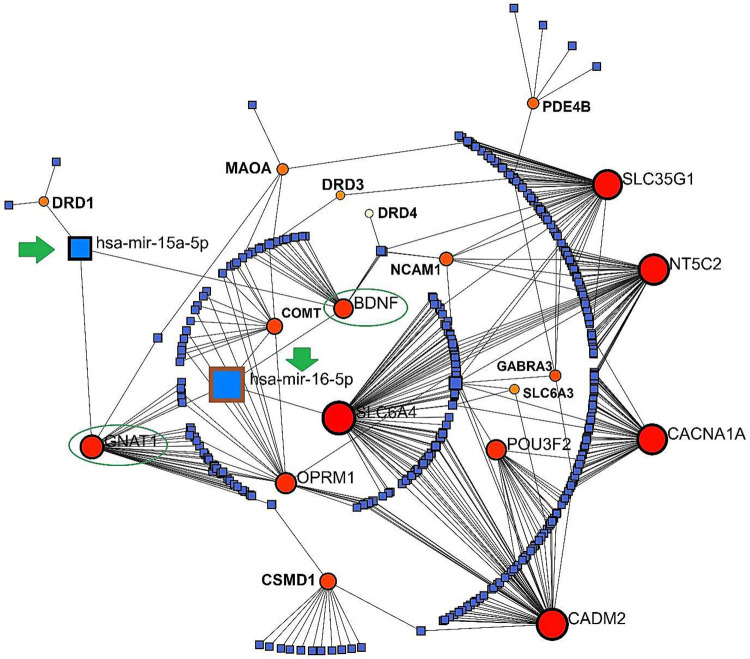
GMIs visualized by NetworkAnalyst in a concentric model concentrating important interactions. The green arrows signify the best-scored miRNAs (hsa-miR-16–5p and hsa-miR-15a-5p) and the green circles highlight the common genes in association with the best-score miRNAs in this network.

Employing NetworkAnalyst which is based on a R package, a Concentric model predicted the potential GMIs among the SGL members (23 genes). GMIs showed 2 highly potential miRNAs which can link GARS genes into Cannabis genes including **hsa-miR-16–5p** and **hsa-miR-15a-5p**. The common genes which both miRNAs have linked with them were ***BDNF*** and ***GNAT1***. Importantly, in this GMI network, the gene member with the highest degree of betweenness (interactions) was *SLC6A4* with 78 interactions (Figure 5).

##### TF-miRNA coregulatory interactions

**Figure 6.**
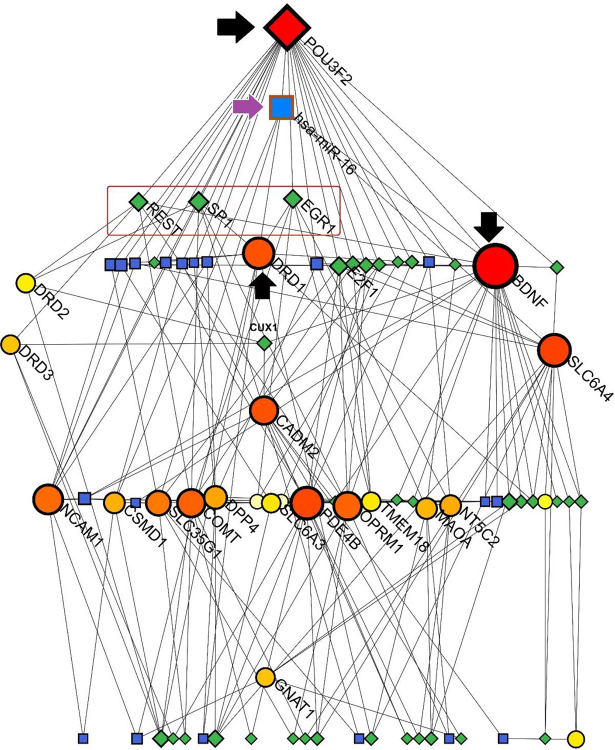
Sugiyama model of TF-miRNAs co-regulatory interactions among the SGL members. The most interacted proteins are clarified larger than others; also, green rhombuses and blue squares refer to Transcription factors (TFs) and miRNAs, respectively.

According to a Sugiyama model by NetworkAnalyst program, results from the NetworkAnalyst tool exposed a connected network among the SGL members, miRNAs and Transcription Factors (TFs). Notably, *POU3F2* and *BDNF* were the major genes with the highest degree of betweenness (23 and 22, respectively). SP1 (degree of betweenness = 7), REST (degree of betweeness = 6), and EGR1 (degree of betweeness = 6) were the most important TFs in this network. Interestingly, hsa-miR-16 was presented as the top-scored miRNAs interacting with *POU3F2, BDNF*, and *DRD1*. DRD1 seemed to have a critical role in this network due to its interactions with SP1 and EGR1 as TFs and hsa-miR-16 as a bridge between TFs and miRNAs. Additionally, *DRD2* had interactions with SP1 and REST transcription factors (Figure 6).

#### Protein-Chemical Interactions (PCIs)

**Figure 8.**
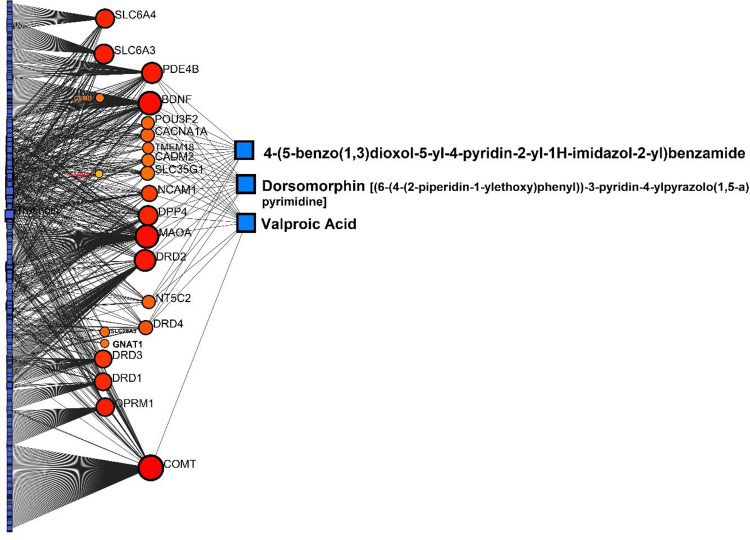
PCIs in a linear bi/tripartite model utilizing NetworkAnalyst and SGL members revealed important interacted compounds. Bigger red circles mean the gene with higher degree of betweeness (interactions). As it is obvious, Valproic acid is among the best-scored predicted for this PCI network.

Along with the in-depth silico analyses that we previously accomplished and described, we further performed PCIs to enhance information and confirmation. PCIs suggested three chemicals with high betweeness degrees including Valproic acid, Dorsomorphin [(6-(4-(2-piperidin-1-ylethoxy)phenyl))-3-pyridin-4-ylpyrazolo(1,5-a)pyrimidine], and 4-(5-benzo(1,3)dioxol-5-yl-4-pyridin-2-yl-1H-imidazol-2-yl)benzamide (all showed 13 interactions). Valproic Acid as the top compound, was linked with *BDNF, DRD4, COMT, MAOA, DPP4, PDE4B, POU3F2, CACNA1A, TMEM18, CADM2, SLC35G1, NCAM1*, and *NT5C2* (Figure 8).

##### Enrichment Analysis (EA)

We piloted an enrichment analysis for systems biology investigation(s) which in turn, was divided into three main classifications including Pathway Analysis, Ontology Analysis, and Disease-Drug Analysis by the Enrichr tool. In this section, we prioritized the SGL members according to their adjusted p-values (q-values) documented from different databases, such as KEGG, Reactome, GO, DisGeNET and GeDiPNET.

#### Pathway Analysis

Table 4 displays pathway analysis of SGL members as a sub-categorized analysis of Enrichment Analysis. In fact, following combining and prioritizing the candidate genes according to the most significant q-values, results from the KEGG database revealed that dopaminergic synapse is the most significant pathway (q-value= 6.03E-11; p-value=1.31E-12; OR=85.39). This finding was confirmed by Reactome as well (q-value= 7.63E-10) (Table 4). Remarkably, Cocaine addiction showed up in the second place and Morphine addiction as third and even alcoholism. Suggestive of Cannabis Use Disorder (CUD) and polysubstance abuse.

#### Ontology Analysis

Table 5 specifies our Ontology analysis, highlighting the significance of Dopamine Metabolic Process (GO:0042417) as the most significant biological process (q-value= 9.77E-12; p-value= 3.053e-14; OR= 16867.35). Additionally, Neuron Projection (GO:0043005) (q-value= 7.49E-05) and Monoamine Transmembrane Transporter Activity (GO:0008504) (q-value=0.004) were significant in the GO Cellular component and GO Molecular Function categories, respectively.

##### Disease-Drug Assessments (DDAs)

To find the manifesting role(s) of SGL members, we conducted another Enrichment Analysis based on disease/drug-based databases including GeDiPNet and DisGeNET. DDAs revealed that the most significant manifestation predicted to be at the highest risk of incidence is Heroin Dependence (q-value= 2.05E-19; p-value= 1.30E-22; and OR= 358.15) [ see reference 69–71 and epigenetics]. The second and third significant manifestations were Amphetamine-Related Disorders (q-value= 6.68E-19) and Autistic Disorder (q-value= 2.17E-18) based on DisGeNET, respectively (Table 6).

#### Pharmacogenomics (PGx) Analysis

In this step, we aimed to improve our findings by employing PGx data for the SGL members based on the formerly described findings. Specifically, we searched for all PGx annotations for each refined gene. Accordingly, 17 genes showed 1,498 PGx annotations, among them, 607 annotations had a significant association with a clinical manifestation. These 17 genes were *DRD1, DRD2, DRD3, DRD4, MAOA, COMT, SLC6A3, SLC6A4, OPRM1, NCAM1, CADM2, BDNF, CSMD1, NT5C2, SLC28A3, PDE4B*, and *CACNA1A*

It should be considered that, these pharmacogenes may have associated annotations with certain manifestations related to Cannabis as depicted in table 7, but not many. thus, we decided to primary report Heroin-associated PGx annotations due to its high prevalence risk predicted in the DDA section.

**Table:** 

GARS GENES AND RISK ALLELE	ASSOCIATION WITH CANNABIS USE MISUSE STUDIES	TOTAL ARTICLES LISTED IN PUBMED
Dopamine D1 Receptor (DRD1): rs4532—risk allele G	2	37
Dopamine D2 Receptor (DRD2): rs1800497—risk allele A1	1	187
Dopamine D3 Receptor (DRD3): rs6280—risk allele C (Ser9Gly)	0	63
Dopamine D4 Receptor (DRD4): rs1800955—risk allele C (48bp repeat VNTR)	0	18
Dopamine Transporter Receptor (DAT1): SLC6A3 3′-UTR—risk allele A9 (40bp repeat VNTR)	0	17
Catechol-O-Methyltransferase (COMT): rs4680—risk allele G (Val158Met)	9	661
μ-Opioid Receptor (OPRM1): rs1799971—risk allele G (A118G)	3	177
γ-Aminobutyric Acid (GABA) A Receptor, β−3 Subunit (GABRB3): CA repeat—risk allele 181	0	2
Monoamine Oxidase A (MAO-A): 3′ 30bp VNTR -risk allele 4R DNRP	0	11
Serotonin Transporter Receptor (5HTT) Linked Promoter Region (5HTTLPR) in SLC6A4: rs25531—risk allele S′	0	9
**TOTTAL**	**15**	**1,182**

These PGx annotations can be considered for future PGx studies in association with Cannabis dependence. All of the PGx details are described in Table 8.

Interestingly, final PGx investigations are completely in favor of all findings of the current study because of highlighting the PGx results linked to *DRD2, DRD1, BDNF*, and *OPRM1* genes as the candidate pharmacogenes for Cannabis dependence.

### 3.4 Recommendations

Khalsa et al.(112) emphasize the need for caution due to the considerable variability in CBD content and the potential for adulteration with newly identified cannabinoids, many of which are structurally similar to either CBD or THC.(135) These variations can significantly affect the safety and efficacy of cannabinoids marketed as medicines. An evidence-based approach is crucial, especially when dealing with substances that remain illegal under U.S. federal law.

Clinicians, particularly those specializing in addiction medicine, should carefully weigh the risks and benefits when considering cannabinoid-based treatments, including medical marijuana. Before prescribing unapproved cannabinoids, it is essential to ensure that patients have explored other evidence-supported treatment options where appropriate.(136) The authors advocate for a systematic and rigorous investigation of cannabinoids like CBD as potential therapeutic agents, rather than promoting or marketing them prematurely without the necessary approval through FDA-recommended drug development protocols.

These protocols include comprehensive studies on pharmacological effects, mechanisms of action, pharmacokinetics and pharmacodynamics, drug-drug interactions, and long-term safety. Such studies must adhere to good manufacturing practice (GMP) to ensure standardized active ingredient concentrations, along with good laboratory practice (GLP) and good clinical practice (GCP) standards, particularly for conducting Phase I, Phase II, and pivotal Phase III clinical trials targeting specific clinical conditions.

Given the high costs associated with clinical research and FDA-required trials, it remains uncertain whether pharmaceutical companies will invest in developing CBD or other cannabinoids for a broad range of clinical uses. THC, due to its potent psychoactive effects and associated risks, is unlikely to be developed further as a therapeutic agent. However, CBD continues to show promise for treating several clinical conditions, most notably its current FDA-approved use for two rare forms of childhood epilepsy. Additionally, the combination of CBD and THC, as in Sativex, has been approved in several countries outside the U.S. for treating multiple sclerosis-related spasticity.

The National Academy of Sciences(137) and the American Psychiatric Association ((138)) have both emphasized the need for more basic and clinical research before cannabinoids like CBD can be approved by regulatory bodies such as the U.S. Food and Drug Administration (FDA) for wider clinical use.

The key takeaway is that, at present, none of the other cannabinoids discussed should be considered as formal treatments for unapproved clinical conditions. Further research and investigation are essential before any such cannabinoids can be endorsed as medicines.

### 3.4 Pro-Dopamine Regulation Solution

Willuhn et al.(139) demonstrated that the misuse of cocaine, as well as non-substance-related addictive behaviors, is linked to a reduction in dopaminergic function. Chronic exposure to psychoactive substances, such as cocaine, has been associated with decreased D2/D3 receptor availability, along with reduced activation in brain regions like the occipital cortex and cerebellum, as observed in a PET study by Tomasi et al.(140) Importantly, addiction specialists may have the ability to address the dopaminergic dysfunction caused by high-potency THC in chronic cannabis users by inducing dopamine homeostasis, which could normalize behavior. Blum’s team developed the first patented Genetic Addiction Risk Severity (GARS) test, which has been linked to clinical outcomes through the Addiction Severity Index (ASI). Over 4,000 patients have been tested using this method, providing a predictive framework for addiction severity.(33,34,141,142)

Addressing the disruptions in neurotransmission caused by chronic exposure to substances like cannabis, opioids, and even behavioral addictions, requires restoring dopamine balance—a process known as “dopamine homeostasis.” Studies have shown that coupling GARS with KB220Z variants, which involve a semi-customized precision Pro-Dopamine Regulation (PDR) tailored to an individual’s genetic profile, can lead to positive outcomes. Blum’s group has documented the beneficial effects of KB220 variants, including the reduction of anhedonia, in more than 36 peer-reviewed clinical trials, some of which included triple-blind placebo-controlled designs.(10,143)

These findings suggest that precision medicine approaches like GARS combined with PDR may hold promise for treating dopaminergic dysfunction in addiction. [See Figure 4]

In adolescents with chronic cannabis use, the goal of treatment should be to enhance brain reward functional connectivity—measured by the synchronization of blood-oxygen-level-dependent (BOLD) signals between brain regions—and improve connectivity volume assessed through voxel-based morphology (VBM). This would help mitigate depression-like symptoms such as anhedonia, as well as address stress-related anti-reward symptoms associated with drug dependence. While we have yet to test cannabis directly, our studies using fMRI in both naïve animals (11) and heroin-abstinent individuals(144) demonstrated BOLD activation of dopaminergic reward pathways and increased dopamine neuronal firing when using KB220Z. These findings provide preliminary evidence for dopaminergic activation in reward circuits.

Millions of people globally face the daily challenge of overcoming addiction to substances, including cannabis. The neuroscience community is making significant strides in understanding brain reward circuitry through advanced molecular-genetic techniques in both animal and human studies. This research is shedding light on the neural mechanisms underlying addiction, including dopamine dysregulation. However, there is ongoing debate regarding the best clinical approach to managing dopamine imbalances to prevent and treat addictive disorders, such as Cannabis Use Disorder (CUD).

An alternative approach could involve two treatment phases: an initial brief blockade of dopamine receptors followed by long-term dopaminergic upregulation. The goal of such treatment would be to restore brain reward functional connectivity and alleviate stress-related anti-reward symptoms commonly seen in addiction. The use of the Genetic Addiction Risk Score (GARS) allows for the identification of reward deficiency phenotypes, and “Precision Addiction Management” (PAM)^®^—customized neuro-nutrient supplementation based on GARS results—could help achieve dopamine homeostasis.(145) Behavioral interventions, such as Awareness Integration Therapy (AIT), could further support this approach.(146)

A case series by Fried et al.(147) highlights an example of personalized medicine through the use of GARS-guided treatment. A 34-year-old female with a history of cannabis abuse and alcoholism was genotyped using the GARS test, revealing a hypodopaminergic risk. She received a pro-dopamine regulator tailored to her genetic profile, which included KB220Z, and subsequently achieved recovery from Substance Use Disorder (SUD). She experienced significant improvements in social interactions, economic status, and overall well-being, along with reduced symptoms of depression. Urine drug tests confirmed her abstinence over a two-month period. Her family members, who also underwent GARS testing and received neuro-nutrient interventions, showed behavioral improvements, illustrating the multi-generational impact of personalized, DNA-guided treatment.(148)

Manza et al.(149) have demonstrated that chronic cannabis use is associated with alterations in resting-state brain function, particularly in dopaminergic areas involved in psychosis, habit formation, and reward processing. This raises the possibility that GARS-guided precision treatments with KB220Z could help restore normal reward processing and connectivity in individuals with cannabis use, especially in adolescents and other high-risk populations.

## 4.0 Summary

Many U.S. states now permit the medical and recreational use of cannabis, leading to increased interest and research into cannabinoid products as well as the potential risks associated with cannabis use, addiction, and intoxication. With marijuana in its plant form, the variety of compounds present can interact with CNR1 receptors in ways that may reduce some of THC’s effects. While this mechanism appears plausible, Vallee et al. (150) may have overstated the potential of CNR1 receptor blockade as a novel approach for treating cannabis intoxication and addiction.

We urge caution in pursuing CNR1 receptor blockers as a treatment strategy, given the historical example of Rimonabant (SR141718), a CNR1 receptor antagonist that was withdrawn from the market due to severe side effects, including mood disturbances and suicidal ideation (151). The FDA also rejected similar drugs because of these risks. Given the well-documented dangers, it is crucial to question the wisdom of revisiting such treatment strategies.(151, 152). Along these lines others have also suggested risks in overusing Cannabis and its abuse (153).

Finally, it is worth mentioning that George Koob, the current Director of the NIAAA, has characterized addiction as a “reward deficiency disease” and a “stress surfeit disease.(23)”

According to the novel findings of the bioinformatic part of the current paper, we clearly described our strategy of analyses and the rationale behind it. Briefly, by collecting all the GWAS data about ‘Cannabis’ term updated on the 5^th^ of June 2025 in GWAS atlas databank, then refining and performing meta analyses and meta-meta analyses, we presented a candidate gene list (SGL) containing 23 unique genes. GARS genes were shown to have strong associations with Cannabis genes in various networks including PPIs, GMIs, TF-miR CoRegIs, and PCIs.

In fact, the utilization of these networks revealed and highlighted some genes such as *DRD2, BDNF, DRD1, GNAT1, SLC6A4*, and *POU3F2* to significantly interact with cannabis. These networks also predicted other biological molecules like miRNAs and TFs showing significant roles correlated with our SGL members like hsa-miR-16–5p and SP1, REST, and EGR1. Additionally, based on the findings from Systems biology analyses, dopaminergic pathway(s) was the top-scored pathway along with heroin dependence as the most plausible manifestation from the interplay among the SGL members. It is of some interest that utilizing this approach, other relatively unknown related systems like ***Negative Regulation Of Protein Secretion*** also showed up.

Lastly, PGx findings suggested PGx annotations associated with Heroin dependence as a strong clinical candidate for future association studies on Cannabis dependence, AT first glance this could be considered surprising, but it seems to eloquently fit well with previously published early epigenetic investigations from Blum’s group in 1980 (69) and confirmed by Hurd’s group in 2014 (70) showing significant increased sensitivity to enkephalins and heroin respectively. The take home message more specifically suggested herein is that risk polymorphisms particularly with, *BDNF, DRD1, DRD2*, and *OPRM1* are highly recommended for future PGx-based investigations on Cannabis.

## 5.0 Conclusion

One challenge facing the scientific community is the growing legalization of cannabis products across various U.S. states. We support reform efforts that focus on either decriminalization or restrictive legalization, particularly with regard to controlling legal limits of THC. As with other psychoactive substances, we hypothesize that chronic use of high-THC cannabis, whether in the form of wax, smoke, or vapor, contributes to brain reward dysfunction by disrupting neurotransmission and causing hypodopaminergia. This imbalance may lead to both substance-related (heroin) and behavioral addictions as well as psychosis.

To reiterate, according to the strong validated predictions of this paper, we suggest that the genetic and epigenetic mechanisms involving Cannabis Dependence might be very similar and overlapping with Heroin Dependence. Interestingly, previous epigenetic results from the laboratories of both Blum’s and Hurd’s groups reveal the epigenetic F1 generational augmented sensitivity and rewarding effects of prenatal utilization of THC for opioids (enkephalins, morphine, heroin etc.). Finally, we believe that our top-scored genes, proteins, miRNAs, TFs, and PGx annotations (PGx variants) should not be ignored and with required more clinical data and intensive investigation can shed a light on uncovering the true Cannabis Use Disorder (CUD) neurobiological phenotypic understanding and measurable genetic and epigenetic endophenotype.

In fact, currently there is a clinical trial (supported by NIDA) regarding CBD to determine if CBD can be used to treat CUD. Further, cannabis in any form that is not approved by the FDA or any other regulatory body, we don’t suggest its use for that clinical condition.

To address the anhedonia and other negative effects induced by chronic THC and other psychoactive drugs, we propose a combined approach that includes genetic risk testing and pro-dopamine regulation. This strategy could help restore dopamine balance and mitigate the long-term effects of high-THC exposure.

## Figures and Tables

**Figure 1:**
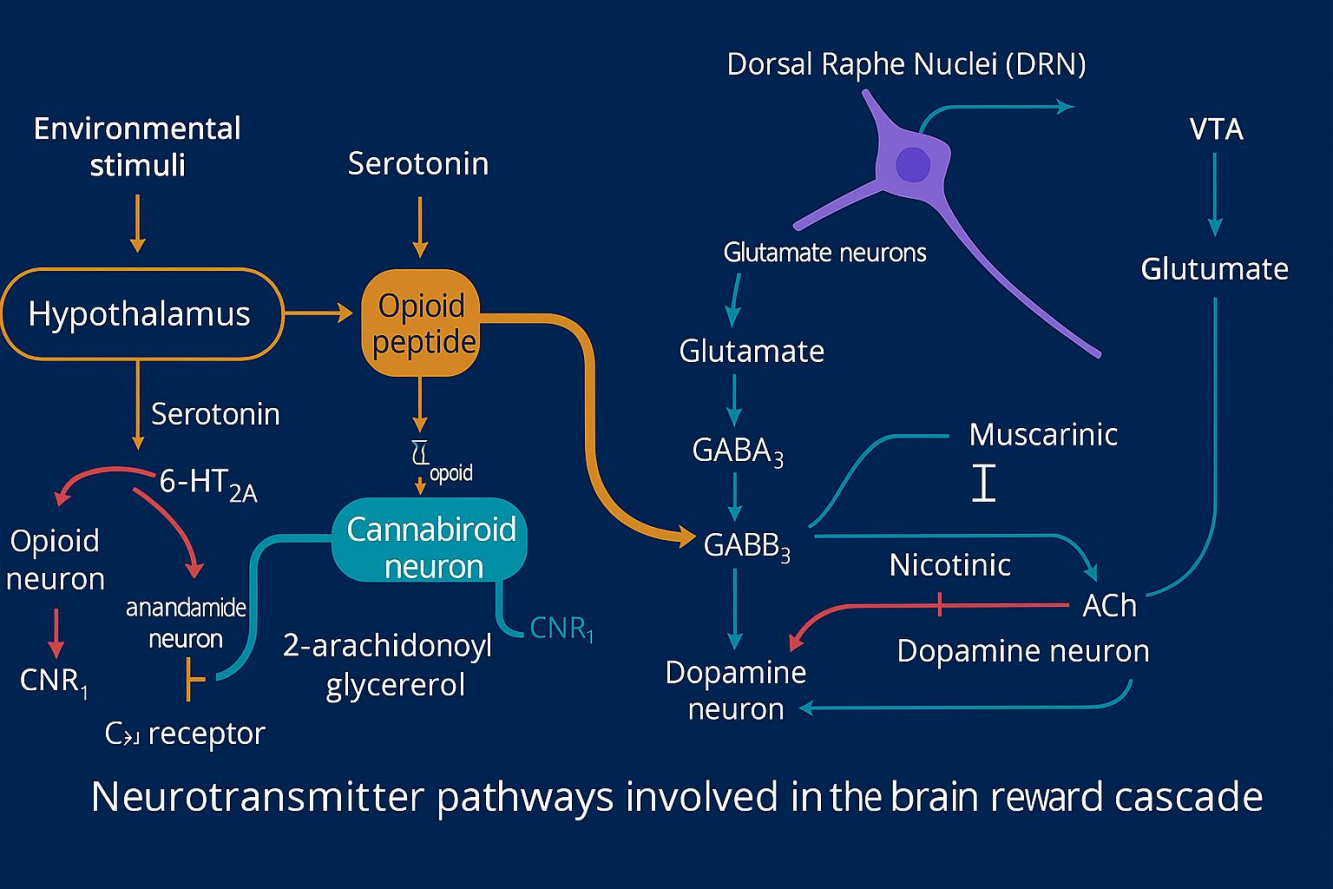
Brain Reward Cascade (BRC): Region-by-region schematic of transmitter–receptor interactions. The diagram depicts a stepwise cascade linking Hypothalamus → Substantia Nigra (SN) → Dorsal Raphe Nuclei (DRN) → Ventral Tegmental Area (VTA) → Nucleus Accumbens (NAc). Serotonin (5-HT) released from the hypothalamus (via 5-HT2A) drives opioid peptide release. In the SN, opioid peptides engage μ-opioid receptors (inhibiting GABA_A neurons, e.g., via enkephalins) and δ-opioid receptors hat promote endocannabinoid signaling (anandamide, 2-AG) acting at CNR1 to further inhibit GABA_A neurons (net disinhibition of downstream output). DRN glutamatergic input (via GLU M3) also contributes to GABA disinhibition in the SN. Disinhibited GABA_Aneurons then suppress the glutamatergic drive to the VTA through GABA_B3 signaling, while VTA glutamateactivates NMDA receptors on dopamine (DA) neurons projecting to the NAc, culminating in DA release (reward signal). In the NAc, acetylcholine (ACh) modulates muscarinic (M) and nicotinic (nAChR) receptors (muscarinic inhibition; nicotinic facilitation), shaping the final reward output. Arrow conventions: red = excitatory; blue = inhibitory; green = modulatory. Abbreviations: 5-HT = serotonin; 2-AG = 2-arachidonoylglycerol; CNR1 = cannabinoid receptor 1; DA = dopamine; ACh = acetylcholine.

**Figure 2.**
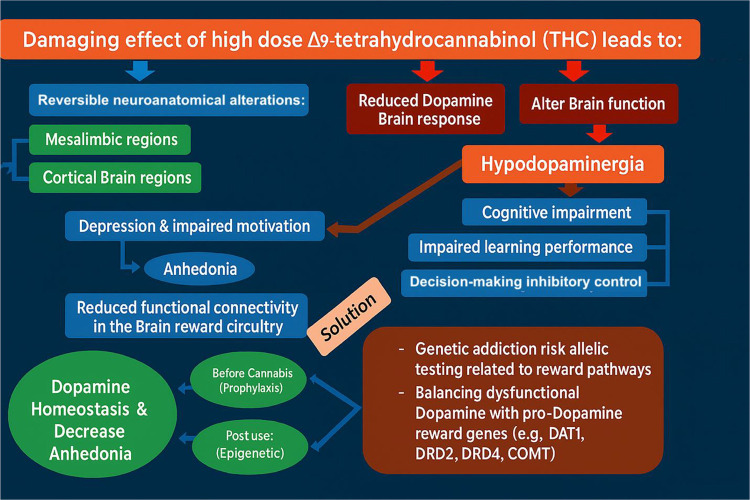
Proposed management model for high-potency cannabis use and anhedonia (adapted with permission [5]). Schematic illustrating a biphasic strategy for Reward Deficiency Syndrome (RDS) behaviors in the context of chronic exposure to high-Δ9-tetrahydrocannabinol (Δ9-THC) products (e.g., gummies, vaping), which elevate the risk of dopamine dysregulation and reward-circuit impairment. Phase 1 employs short-term dopamine blockade to dampen cue-reactivity, cravings, and compulsive use. Phase 2 transitions to sustained dopaminergic upregulation—aimed at restoring reward-circuit tone and reducing anhedonia—via pro-dopaminergic, behavioral, and rehabilitative interventions. The model emphasizes timing, sequencing, and individualized risk assessment to rebalance mesolimbic function after high-potency cannabis exposure. Abbreviations: Δ9-THC, Δ9-tetrahydrocannabinol; RDS, Reward Deficiency Syndrome.

**Figure 3:**
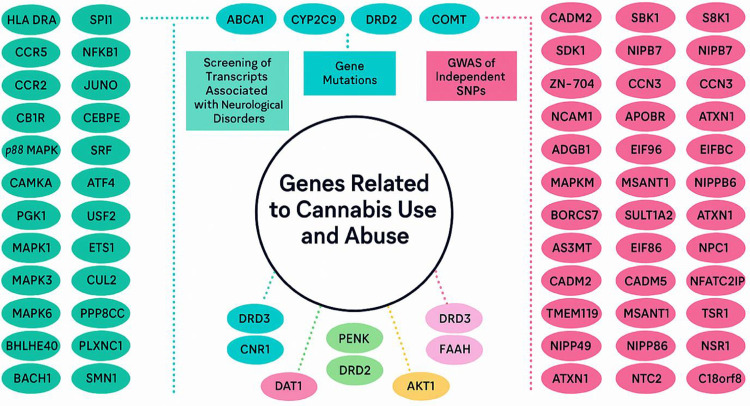
Genes implicated in cannabis use and Cannabis Use Disorder (CUD) overlap with pathways linked to schizophrenia, depression, anxiety, and Reward Deficiency Syndrome (RDS), including substance use disorders. Clarifying how genetic and epigenetic variation modulates cannabinoid signaling is pivotal for mechanistic research, targeted therapeutics, and the development of purified cannabinoid compounds suitable for FDA evaluation. Deeper insight into these determinants will enable more effective treatments and advance personalized medicine (reproduced with permission (5)).

**Figure 1.**
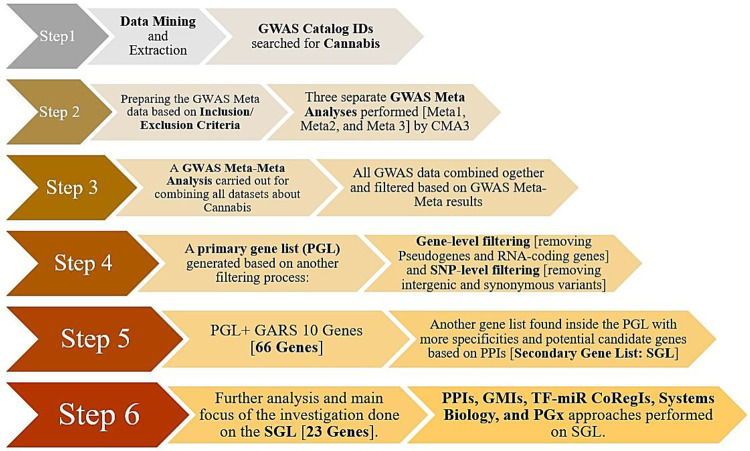
this illustration reveals a flowchart of the analysis’s strategy employed in this study. Abbreviations are as follows: GWAS: Genome-Wide Association Studies; CMA3: Comprehensive Meta Analysis 3; PGL: Primary Gene List; SGL: Secondary Gene List; PPIs: Protein-Protein Interactions; GMIs: Gene-miRNA Interactions; and TF-miR CoRegI: Transcription Factor- miRNA Co-Regulatory Interactions.

**Figure 2.**
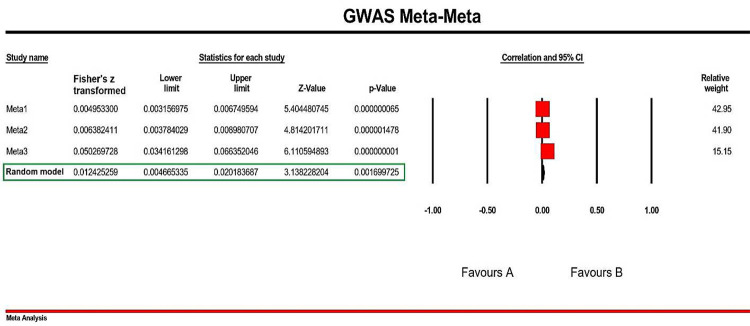
Forest Plot of GWAS Meta-Meta Analyses performed on 3 separate Meta groups including Meta1 (Cannabis Use), Meta2 (Cannabis Dependence), and Meta3 (Cannabis Dependence Measurement). Effect size [partial correlation coefficients (PCC) using Fisher’s z transformation] was set on Random status with 2-tailed and positive effect direction. Obviously, all included Meta data are in 0 range between the Favors A and B ranges. The relative weight of Meta1 and Meta2 are so close together, and as such unfortunately just Meta3 represented low relative weight resulted from its low sample size in comparison with the other Meta data.

**Figure 3.**
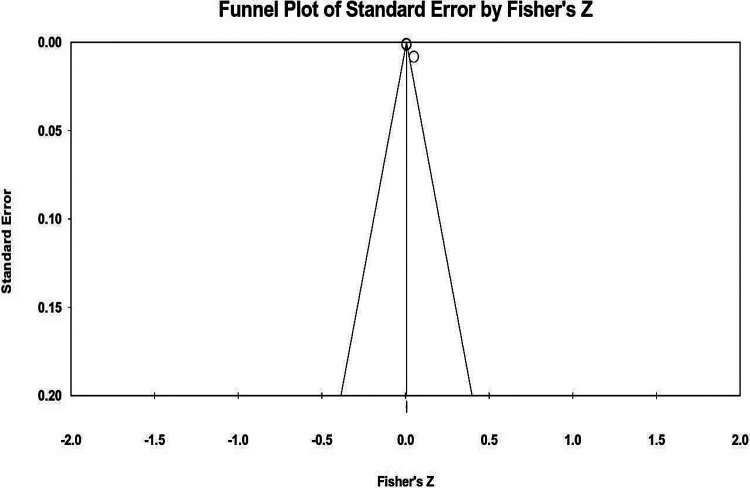
Funnel Plot of GWAS Meta-Meta Analysis envisaged by CMA3 displaying the likelihood of publication bias based on the Standard Error by Fisher’s Z. Noticeably, there is no publication bias approving the validity of this Meta-Meta Analyses.

**Figure 4.**
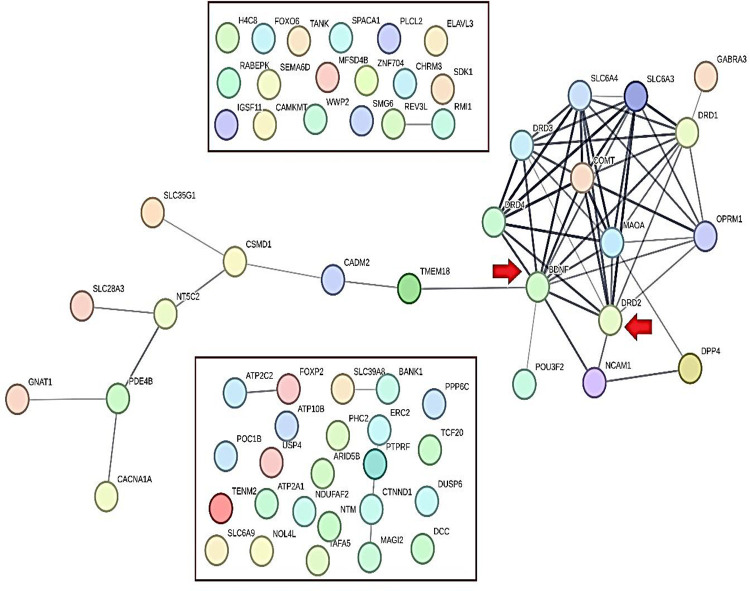
Illustrates STRING-MODEL of the 66 Pre-Primary Gene List (Pre-PGL) linked to Cannabis as predicted by the Protein-Protein Interactions (PPIs) network. There are two rectangular zones separated from the main network by red indicating the unconnected protein-coding genes. Also, red arrows highlight the potential roles of DRD2 and BDNF as linking members of GARS genes and Cannabis candidate genes.

**Figure 4:**
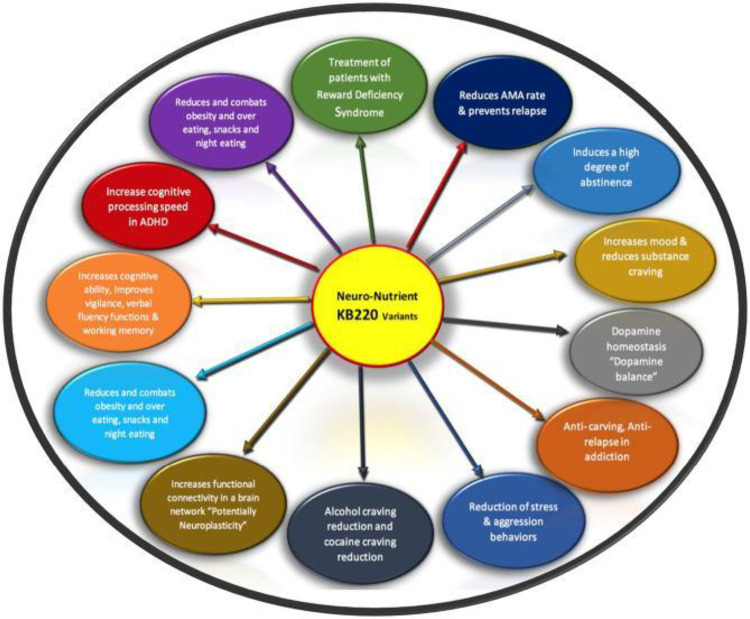
Schematic representation of the benefits of KB220 variants for Reward Deficiency Syndrome (RDS). Adapted from Blum, 2020, with permission. The most recent formulation of KB220Z in powdered form includes a variety of key ingredients designed to support dopaminergic function. The ingredients are as follows: Vitamin B6 (10 mg, 500% of Daily Value), Thiamine (15 mg, 1,033% of Daily Value), and Chromium polynicotinate (200 mcg, 166%). Additionally, a fixed dose of Synaptose, a blend of amino acids and herbal extracts, is included. Synaptose contains DL-Phenylalanine, L-Tyrosine, Passion-Flower Extract, along with a complex of Arabinogalactans, N-Acetylglucosamine, Astragalus, Aloe Vera, Frankincense Resin, White Pine Bark Extract, and Spirulina. Other active components include Rhodiola, L-Glutamine, 5-Hydroxytryptophan (5-HTP), Thiamine Hydrochloride, Pyridoxal-5-phosphate, Pyridoxine HCl, CoQ10, NADH, and N-Acetyl Cysteine (NAC).(11) This formulation was manufactured by Cephram, Inc. (New Jersey).

**Table 1:** Listing of products by manufacturer

Product	Manufacturer	Cannabis-related properties	Potential/approved indication	Current approval status
Dronabinol/Marinol	Solvay pharmaceuticals	Synthetic THC	Chemotherapy-related nausea/vomiting; appetite stimulation in AIDS patients	Approved
Nabilone/Cesamet	Valeant Pharmaceuticals International	Synthetic cannabinoids like THC	Chemotherapy-related nausea/vomiting	Approved
CBD/Epidiolex	GW pharmaceuticals	CBD	Epilepsy/LG-Dravet syndrome	Approved
Nabiximols/Sativex	GW pharmaceuticals	CBD + THC in 1:1 ratio, oral mouth spray	MS-associated Neuropathic pain and spasticity	Approved in 28 countries but not in the US
Dexanabinol	Solvay pharmaceuticals	Synthetic non-psychotic cannabinoid that blocks NMDA receptors and COX-2 cytokines and chemokines	Neuroprotective for use after cardiac surgery, regain memory and brain function following traumatic brain injury, possible anti-cancer	NOT approved due to failed efficacy
CT-3 (ajulemic acid)	Indevus pharmaceuticals	Synthetic, potent analog of THC metabolite, THC-11-oic acid	MS-associated spasticity; anti-inflammatory for arthritic pain	NOT approved
Cannabinor (PRS-211,275)	Pharmos	Synthetic cannabinoid that binds to CB2 receptors	Anti-inflammatory for chronic neuropathic pain, bladder control	NOT approved
HU-308	Pharmos	Synthetic cannabinoid that binds to CB2 receptors	Anti-inflammatory; hypertension	NOT approved
HU-331	Cayman chemicals	Synthetic cannabinoids that bind to CNR1 and CB2 receptors	Memory, weight loss, appetite stimulant, neurogeneration, tumor surveillance, analgesia, inflammation	NOT approved
Rimonabant/Acomplia	Sanofi/Aventis	Synthetic chemical blocks endocannabinoids	Anti-obesity (appetite suppression)	NOT approved; Sanofi withdrew due to adverse effects of suicidal ideations
Trainband/MK-0364	Merck	Synthetic chemical targets appetite controlling receptors; acts via CNR1R receptors	Anti-obesity	NOT approved; Merck stopped further development due to ADRs like anxiety and depression

FDA-approved and non-approved Cannabis-based Pharmaceuticals (Pharmaceutical Drugs Based on Cannabis—Medical Marijuana—ProCon.org; accessed 07,23,2022) (FDA Regulation of Cannabis and Cannabis-Derived Products, Including Cannabidiol (CBD) | FDA; accessed 07,23,2022). (Reproduced with permission.(112); ADRs = adverse drug reactions)

**Table 2:** Therapeutic Potential of Cannabinoids Based on Research Reviewed

Cannabinoid	Pharmacologic activity	Potential Indication
Cannabidiol	Anti-inflammatory, neuroprotective, antioxidant, cardioprotective, anti-angiogenesis	Anxiety (94*), Alzheimer’s, autism, depression (61*), epilepsy/seizures (44*), inflammation, multiple sclerosis (7*), pain (64*), Parkinson’s disease (4*), trauma, colitis, skin disorders, substance use disorders (15*)
Cannabichromene	Anti-inflammatory, neuroprotective	Epilepsy, skin disorders
Cannabidivarin	Anti-inflammatory, neuroprotective	Autism, epilepsy, skin disorders,
Tetrahydrocannabivarin	Anti-inflammatory, neuromodulatory, antioxidant, cardioprotective	Cancer, cardiovascular dysfunction, diabetes, neuropathy, nephropathy, pain, retinopathy
Cannabigerol	Anti-inflammatory, neuroprotective, anti-proliferative	Inflammation, pain, multiple sclerosis, colitis, skin disorders, cancer

The clinical conditions summarized above are where individual cannabinoids have shown some clinical evidence supporting further development as a therapeutic;

* = number of clinical trials investigating clinical conditions registered at: https://clinicaltrials.gov. Reproduced with permission.(112)

**Table 1.** Tools, validations, and references of all in-depth silico databases and systems biology approaches applied in this investigation.

Level	Database/Tool	Site	Software (version)	References
GWAS data mining	GWAS catalog	https://www.ebi.ac.uk/gwas/home	EMBL-EBI 2024	116
GWAS Meta Analysis	CMA	https://meta-analysis.com/	CMA (3)	117
GWAS Meta-Meta Analysis	CMA	https://meta-analysis.com/	CMA (3)	117
PPIs	STRING-MODEL	https://string-db.org/	STRING (12.0)	118
GMIs	miRTarBase v9.0	https://mirtarbase.cuhk.edu.cn/~miRTarBase/miRTarBase_2019/php/index.php	NetworkAnalyst (3.0)	119
GNR	TF-coregulatory Interactions	http://www.regnetworkweb.org/	NetworkAnalyst (3.0)	119
EA	Pathway Analysis	https://maayanlab.cloud/Enrichr/	Enrichr	120
	GO	https://maayanlab.cloud/Enrichr/	Enrichr	120
	DDA	https://maayanlab.cloud/Enrichr/	Enrichr	120
PGx	VAA	https://www.pharmgkb.org/	PharmGKB	121, 122

The abbreviations are as follows: GWAS: Genome-Wide Association Study; CMA: Comprehensive Meta-Analysis; PPIs: Protein-Protein Interactions; GMIs: Gene-miRNA Interactions; GRNs: Gene Regulatory Networks; EA: Enrichment Analysis; GO: Gene Ontology; DDA: Diseases Drugs Assessment; VAA: Variant Annotation Assessment.

**Table 2.** Characteristics of entry data in CMA3 from GWAS atlas.

Meta group	Trait	GWAS CID	Unique Studies	Sample Size	Primary Unique Mapped Genes with P<E-08
Meta1	Cannabis Use	EFO_0007585	7	1,190,454	28
Meta2	Cannabis Dependence	EFO_0007191	5	568,944	37
Meta3	Cannabis Dependence Measurement	EFO_0008457	1	14,754	2
Total	-	-	13	1,774,152	67

GWAS refers to Genome-Wide Association Studies and CID mean Catalog ID.

**Table 3.** Statistical scores regarding GWAS Meta-Meta Analyses performed on Meta1, Meta2, and Meta3 datasets.

Statistical method	Measure	Score
Classic Fail-Safe N	Z for Alpha	1.956
Orwin’s Fai-Safe N	Fisher’s z transformation in observed studies	0.005
Kendall’s Tau without continuity correction	Tau	1.00
Kendall’s Tau with continuity correction	Tau	0.66
Egger’s Regression Intercept	Standard Error	0.74
	t-value	8.128
Duval and Tweedie’s trim and fill	Q-value	63.022
	Random Point Estimated	0.004
	Random Point Estimated Lower	0.003
	Random Point Estimated Upper	0.006

All of the scores with details have been derived from the probability in our study bias and indicate the adjusting method of Fisher’s z transformation.

**Table 4.** Pathway analysis of SGL members as a sub-categorized analysis of Enrichment Analysis.

Index	Name	P-value	q-value	OR
KEGG	Dopaminergic synapse	1.31E-12	6.03E-11	85.39
Reactome	Dopamine Receptors	6.64E-12	7.63E-10	4205.47
Reactome	Neurotransmitter Clearance	2.78E-10	1.60E-08	700.74
KEGG	Cocaine addiction	2.33E-09	5.36E-08	125.84
KEGG	Morphine addiction	5.50E-08	8.44E-07	64.25
Reactome	Amine Ligand-Binding Receptors	1.59E-07	6.1E-06	107.63
Reactome	Transmission Across Chemical Synapses	4.66E-07	1.34E-05	26.46
KEGG	Neuroactive ligand-receptor interaction	1.86E-06	1.78E-05	20.69
KEGG	Alcoholism	1.94E-06	1.78E-05	30.38
Reactome	Neuronal System	5.45E-06	0.000125	17.06
Reactome	Signaling by GPCR	9.91E-06	0.00019	12.07
Reactome	G Alpha (I) Signalling Events	2.54E-05	0.000417	17.57
Reactome	Class A 1 (Rhodopsin-like Receptors)	3.27E-05	0.000469	16.64
KEGG	Amphetamine addiction	6.62E-05	0.000508	45.25
KEGG	Synaptic vesicle cycle	9.55E-05	0.000573	39.8
KEGG	cAMP signaling pathway	9.97E-05	0.000573	19.63
Reactome	GPCR Downstream Signalling	6.25E-05	0.000795	10.89
Reactome	Ribavirin ADME	6.91E-05	0.000795	211.3
KEGG	Parkinson disease	0.000172	0.000881	16.96
KEGG	Serotonergic synapse	0.000286	0.001317	27.09

q-value and OR refer to Adjusted p-value and Odds Ration, respectively.

**Table 5.** Gene Ontology (GO) analysis of candidate genes in biological, cellular, and molecular processes.

Index	Name	P-value	q-value	OR
GO Biological Process	Dopamine Metabolic Process (GO:0042417)	3.05E-14	9.77E-12	542.01
GO Biological Process	Catecholamine Metabolic Process (GO:0006584)	1.62E-12	2.58E-10	693.37
GO Biological Process	Response To Ethanol (GO:0045471)	4.02E-09	4.29E-07	300.2
GO Biological Process	Prepulse Inhibition (GO:0060134)	1.33E-08	1.06E-06	1498.13
GO Biological Process	Response To Cocaine (GO:0042220)	2.65E-08	1.7E-06	998.7
GO Biological Process	Regulation Of Dopamine Uptake Involved In Synaptic Transmission (GO:0051584)	4.64E-08	2.47E-06	748.99
GO Biological Process	Negative Regulation Of Protein Secretion (GO:0050709)	1.06E-07	3.55E-06	119.95
GO Biological Process	Response To Histamine (GO:0034776)	1.11E-07	3.55E-06	499.27
GO Biological Process	Arachidonate Transport (GO:1903963)	1.11E-07	3.55E-06	499.27
GO Biological Process	Arachidonic Acid Secretion (GO:0050482)	1.11E-07	3.55E-06	499.27
GO Cellular Component	Neuron Projection (GO:0043005)	2.08E-06	7.49E-05	15.45
GO Cellular Component	Dendrite (GO:0030425)	1.19E-05	0.000214	20.66
GO Cellular Component	Axon (GO:0030424)	8.15E-05	0.000978	20.71
GO Cellular Component	Cell Projection Membrane (GO:0031253)	0.000231	0.002076	29.23
GO Cellular Component	Non-Motile Cilium (GO:0097730)	0.00054	0.003885	67.85
GO Molecular Function	Monoamine Transmembrane Transporter Activity (GO:0008504)	6.91E-05	0.004063	211.3
GO Molecular Function	Sodium: Chloride Symporter Activity (GO:0015378)	9.79E-05	0.004063	172.87
GO Molecular Function	G Protein-Coupled Receptor Activity (GO:0004930)	0.000175	0.004436	16.89
GO Molecular Function	Postsynaptic Neurotransmitter Receptor Activity (GO:0098960)	0.000214	0.004436	111.82

q-value and OR mean adjusted p-value and Odds Ratio.

**Table 6.** Disease-Drug Assessments (DDAs) of SGL members according to DisGeNET and GeDiPNeT databases.

Index	Name	P-value	q-value	OR
DisGeNET	Heroin Dependence	1.30E-22	2.05E-19	358.15
DisGeNET	Amphetamine-Related Disorders	1.46E-21	6.68E-19	280.81
DisGeNET	Amphetamine Abuse	1.46E-21	6.68E-19	280.81
DisGeNET	Amphetamine Addiction	1.70E-21	6.68E-19	276.54
DisGeNET	Autistic Disorder	6.90E-21	2.17E-18	82.93
GeDiPNet	Bipolar Disorder	1.13E-20	2.91E-18	80.4
DisGeNET	Bipolar Disorder	2.52E-19	6.60E-17	66.19
DisGeNET	Nicotine Dependence	4.20E-19	8.84E-17	161.14
DisGeNET	Impulsive character (finding)	5.02E-19	8.84E-17	356.09
DisGeNET	Unipolar Depression	5.10E-19	8.84E-17	72.74
DisGeNET	Alcohol or Other Drugs use	5.62E-19	8.84E-17	665.37
GeDiPNet	Mental Depression	2.77E-17	3.55E-15	49.14
GeDiPNet	Mood Disorder	2.49E-15	2.13E-13	70.06
GeDiPNet	Schizophrenia	2.60E-12	1.67E-10	23.85
GeDiPNet	Anxiety Disorder	2.28E-10	1.17E-08	33.24
GeDiPNet	Manic Disorder	2.99E-10	1.28E-08	96.23
GeDiPNet	Cognitive Disorder	1.88E-09	6.89E-08	131.85
GeDiPNet	Minimal Brain Dysfunction	9.59E-09	3.08E-07	233.44
GeDiPNet	Status Marmoratus	1.16E-08	3.31E-07	221.14
GeDiPNet	Anhedonia	1.39E-08	3.57E-07	210.07

q-value stands for adjusted p-value and OR shows Odds Ratio.

**Table 8.** PGx annotations screened among SGL members in accordance with PharmGKB data.

Genes	Variant	Association	P-Value	Drugs	Ref
BDNF	rs16917234	Genotype TT is associated with decreased age at onset of Heroin Dependence due to heroin as compared to genotypes CC + CT.	0.012	heroin	125
BDNF	rs6265	Genotype TT is associated with decreased age at onset of Heroin Dependence due to heroin as compared to genotypes CC + CT.	0.032	heroin	125
DRD1	rs5326	Genotype TT is associated with increased dose of methadone in people with Heroin Dependence as compared to genotypes CC + CT.	0.01	methadone	126
DRD2	rs1076560	Allele A is associated with increased likelihood of Heroin Dependence due to heroin as compared to allele C.	0.021	heroin	127
DRD2	rs12364283	Allele G is associated with increased risk of Heroin Dependence due to heroin as compared to allele A.	8.89E-06	heroin	128
DRD2	rs1799978	Genotype CC is associated with decreased dose of methadone in people with Heroin Dependence as compared to genotype TT.	0.01	methadone	129
DRD2	rs6275	Genotype AA is associated with decreased dose of methadone in people with Heroin Dependence as compared to genotype GG.	0.002	methadone	129
OPRM1	rs10457090	Allele G is associated with increased concentrations of cotinine in people with Heroin Dependence as compared to allele A.	0.004	cotinine	130
OPRM1	rs1074287	Allele G is associated with increased concentrations of cotinine in people with Heroin Dependence as compared to allele A.	0.002	cotinine	130
OPRM1	rs12209447	Allele T is associated with increased concentrations of cotinine in people with Heroin Dependence as compared to allele C.	0.009	cotinine	130
OPRM1	rs1799971	Allele G is associated with increased risk of Heroin Dependence due to heroin as compared to allele A.	0.016	heroin	131
OPRM1	rs2075572	Allele G is associated with increased concentrations of cotinine in people with Heroin Dependence as compared to allele C.	0.03	cotinine	130
OPRM1	rs3778150	Allele C is associated with increased risk of Heroin Dependence due to heroin as compared to allele T.	4.3E-08	heroin	132
OPRM1	rs3778151	Allele C is associated with increased risk of Heroin Dependence due to heroin as compared to allele T.	2.4E-07	heroin	132
OPRM1	rs3778152	Allele G is associated with increased concentrations of cotinine in people with Heroin Dependence as compared to allele A.	0.006	cotinine	130
OPRM1	rs3798676	Allele T is associated with increased concentrations of cotinine in people with Heroin Dependence as compared to allele C.	0.007	cotinine	130
OPRM1	rs3823010	Allele A is associated with increased risk of Heroin Dependence due to heroin as compared to allele G.	2.7E-08	heroin	132
OPRM1	rs495491	Allele G is associated with increased risk of Heroin Dependence due to heroin as compared to allele A.	0.00018	heroin	132
OPRM1	rs510769	Allele T is associated with increased risk of Heroin Dependence due to heroin as compared to allele C.	0.0007	heroin	132
OPRM1	rs511435	Allele T is associated with increased risk of Heroin Dependence due to heroin as compared to allele C.	0.000003	heroin	132
OPRM1	rs524731	Allele A is associated with increased risk of Heroin Dependence due to heroin as compared to allele C.	1.2E-06	heroin	132
OPRM1	rs553202	Allele T is associated with increased concentrations of cotinine in people with Heroin Dependence as compared to allele C.	0.02	cotinine	130
OPRM1	rs562859	Allele C is associated with increased risk of Heroin Dependence due to heroin as compared to allele T.	0.0043	heroin	132
OPRM1	rs563649	Allele T is associated with increased concentrations of cotinine in people with Heroin Dependence as compared to allele C.	0.006	cotinine	130
OPRM1	rs589046	Allele T is associated with increased concentrations of cotinine in people with Heroin Dependence as compared to allele C.	0.001	cotinine	130
OPRM1	rs62638690	Allele T is associated with decreased likelihood of Cocaine-Related Disorders or Heroin Dependence due to cocaine or heroin as compared to allele G.	0.02	cocaine; heroin	133
OPRM1	rs6912029	Allele T is associated with increased concentrations of cotinine in people with Heroin Dependence as compared to allele G.	0.01	cotinine	130
OPRM1	rs7748401	Allele G is associated with increased concentrations of cotinine in people with Heroin Dependence as compared to allele T.	0.006	cotinine	130
OPRM1	rs9479757	Genotype GG is associated with increased severity of Heroin Dependence due to heroin as compared to genotypes AA + AG.	0.008	heroin	134

Ref is the abbreviation of reference.
